# Use of Artificial Intelligence in Drug Development

**DOI:** 10.1001/jamanetworkopen.2024.14139

**Published:** 2024-05-31

**Authors:** Louise C. Druedahl, W. Nicholson Price, Timo Minssen, Ameet Sarpatwari

**Affiliations:** 1 Centre for Advanced Studies in Bioscience Innovation Law (CeBIL), Faculty of Law, University of Copenhagen, Copenhagen, Denmark; 2 University of Michigan Law School, Ann Arbor; 3 Program On Regulation, Therapeutics, And Law (PORTAL), Division of Pharmacoepidemiology and Pharmacoeconomics, Department of Medicine, Brigham and Women’s Hospital and Harvard Medical School, Boston, Massachusetts

## Abstract

This cross-sectional study investigates the scope and breadth of artificial intelligence use in drug development.

## Introduction

Considerable focus has been placed on the health care applications of artificial intelligence (AI). Already, machine learning, a subset of AI that involves “the use of data and algorithms to imitate the way that humans learn”^1^ has been used to predict diseases,^2^ while AI-powered smartphone apps have been developed to promote mental health and weight loss.^3^ Owing in part to such successes, the market for AI in health care has been forecasted to increase more than 1000% between 2022 and 2029, from $13.8 billion to $164.1 billion.^4^

One area of substantial promise is drug development, which is poised to benefit from advances in the use of AI to predict protein folding, molecular interactions, and cellular disease processes.^5^ Successful application of AI to drug development, however, faces several obstacles, including poor model performance caused by nondiverse training data and shortcut learning. Additionally, the often opaque ways that AI systems reach their predictions conflict with regulatory approval frameworks that require a rationale for decision-making. Given these obstacles, we sought to identify the scope and breadth of AI use in drug development.

## Methods

We conducted a cross-sectional study of investigational and approved drugs (n = 102 454) listed in the global research and drug development database Pharmaprojects (Informa, Citeline) on February 11, 2024. Institutional review board approval was not required because this study did not involve human participants, in accordance with 45 CFR §46. We followed the STROBE reporting guideline.

**Figure 1.**
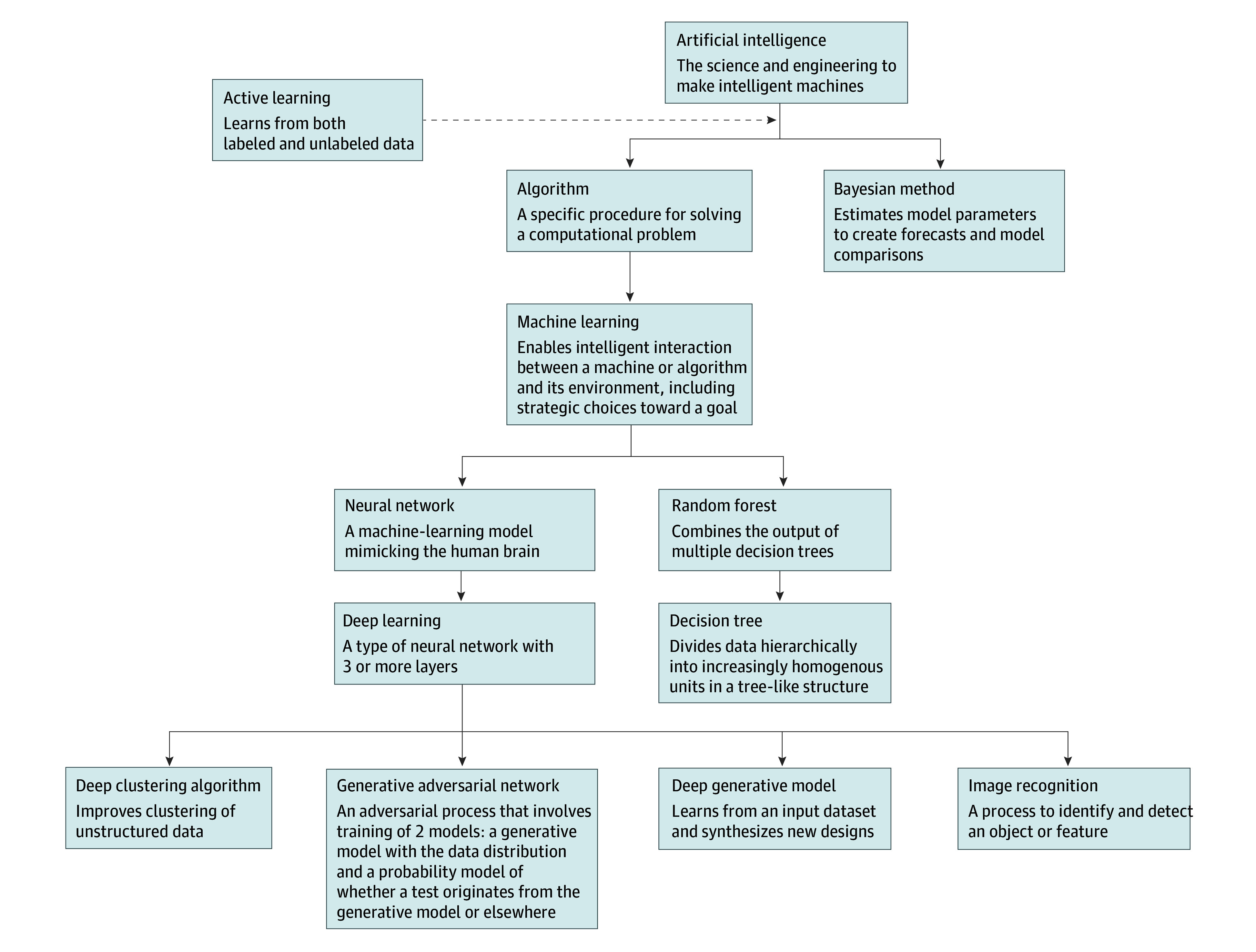
Categories of Artificial Intelligence (AI)

To identify AI-developed drugs, we used AI search terms from Janiesch et al^6^ and the National Library of Medicine’s Medical Subject Headings database (eAppendix in Supplement 1). Automated scans of information for each drug were evaluated. If a drug was described as developed with AI, the type and purpose of AI use were noted. When the type or purpose of AI use could not be determined, additional information was obtained from internet sources. The most specific term identified was used to categorize the AI application (Figure 1). Among AI-developed drugs, descriptive statistics were compiled of AI type, AI purpose, therapeutic area, and development status. Statistical analysis was performed using Excel version 16 (Microsoft) from February to March 2024.

## Results

**Figure 2.**
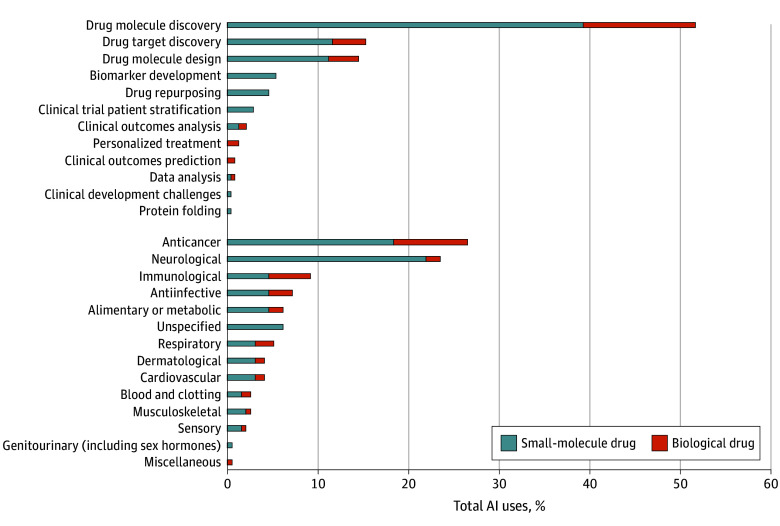
Purpose of Artificial Intelligence (AI) Use

The database search yielded 406 drugs, of which 241 were excluded upon review for no reported use of AI in drug development. AI use was reported in the development of 164 investigational drugs and 1 approved drug. The most frequent types of AI use were machine learning (n = 46 [28%]) and deep learning (n = 28 [17%]). AI was used for 12 purposes, most commonly drug molecule discovery (n = 125 [76%]) (Figure 2). Examples of such use ranged from platform screening of drugs, in which AI was used to analyze molecular images of the effects of drugs on a cell, to deep generative modeling to design virtual novel molecules. Modest AI use was observed for drug target discovery (n = 37 [22%]), including machine learning to find previously unknown connections between genomic, chemical, and clinical data. AI use for clinical outcomes analysis, such as the use of AI-based pattern recognition algorithms to identify correlations between immune responses and patient survival, was more limited (n = 5 [3%]). Regarding therapeutic area, AI use was most common for intended anticancer (n = 52 [32%]) and neurological (n = 46 [28%]) treatments. For the 1 approved drug, which was the stem cell therapy remestemcel-L, a bayesian method was used to estimate the likelihood of obtaining significant results on the primary end point at study completion.

## Discussion

This study found modest use of AI for drug development focused primarily on early-stage applications and on anticancer and neurological therapies. Possible explanations include a lack of high-quality data available in the subsequent stages of drug discovery and uncertain regulatory expectations concerning late-stage AI applications. Study limitations included relying upon public disclosures by drug manufacturers. Ultimately, this study’s results suggest that greater clarity from medicines regulators is needed to guide sponsors over acceptable AI standards and applications to satisfy marketing authorization requirements.

## Notes

Supplement 1. **eAppendix.** Search Terms Used for Data Extraction, Based on Janiesch et al. and MeSH-Terms for Artificial Intelligence

Supplement 2.
Data Sharing Statement
