# Errors in Results

**DOI:** 10.1001/jamanetworkopen.2024.25138

**Published:** 2024-07-03

In the Research Letter titled “Use of Artificial Intelligence in Drug Development,”^1^ published May 31, 2024, 2 percentages in the Results section were given incorrectly. The percentage of drugs for which AI was used for a cancer indication should have appeared as 32% instead of 27%, and the number and percentage of drugs for which AI was used for a neurological indication should have appeared as n = 46 and 28% instead of 24%. This article has been corrected.^1^
