# Supplementary material for: Use of Artificial Intelligence in Drug Development
Source: JAMA Netw Open. 2024 May 31;7(5):e2414139. doi: 10.1001/jamanetworkopen.2024.14139 (PMC11143455; doi:10.1001/jamanetworkopen.2024.14139)
Supplement: Supplement 1. — eAppendix. Search Terms Used for Data Extraction, Based on Janiesch et al. and MeSH-Terms for Artificial Intelligence [file jamanetwopen-e2414139-s001.pdf]

Supplemental Online Content

Druedahl LC, Price WN, Minssen T, Sarpatwari A. Use of artificial intelligence in drug development. *JAMA Netw Open.* 2024;7(5):e2414139. doi:10.1001/jamanetworkopen.2024.14139

eAppendix. Search Terms Used for Data Extraction, Based on Janiesch et al. and MeSH-terms for Artificial Intelligence

This supplemental material has been provided by the authors to give readers additional information about their work.

© 2024 Druedahl LC et al. *JAMA Network Open.*

eAppendix. Search terms used for data extraction, based on Janiesch et al.[1] and MeSH-terms for artificial intelligence.

- Algorithm
- Artificial intelligence
- Autoencoder
- Bayesian method
- Computational intelligence
- Computer heuristics
- Computer reasoning
- Computer vision system
- Decision tree
- Deep learning
- Distributed representation
- Expert systems
- Fuzzy logic
- Image recognition
- Knowledge acquisition (Computer)
- Knowledge representation (Computer)
- Machine intelligence
- Machine learning
- Natural language processing
- Neural network
- Random forest
- Reinforcement learning
- Robotics
- Sentiment analysis
- Supervised learning
- Support vector machine
- Support vector network
- Unsupervised learning

eReference

1. Janiesch C, Zschech P, Heinrich K. Machine learning and deep learning. *Electronic Markets*. 2021;31:685–695. doi:10.1007/S12525-021-00475-2

© 2024 Druedahl LC et al. *JAMA Network Open.*
