# Supplementary material for: Use of Artificial Intelligence in Drug Development
Source: JAMA Netw Open. 2024 May 31;7(5):e2414139. doi: 10.1001/jamanetworkopen.2024.14139 (PMC11143455; doi:10.1001/jamanetworkopen.2024.14139)
Supplement: Supplement 2. — Data Sharing Statement [file jamanetwopen-e2414139-s002.pdf]

Data Sharing Statement

Druedahl. Use of Artificial Intelligence in Drug Development. *JAMA Netw Open*. Published May 31, 2024. doi:10.1001/jamanetworkopen.2024.14139

Data
Data available: Yes
Data types: Data (not involving human participants)
How to access data: The original data set is available upon request to asarpatwari@bwh.harvard.edu
When available: With publication

Supporting Documents
Document types: None

Additional Information
Who can access the data: N/A
Types of analyses: N/A
Mechanisms of data availability: N/A
